# Association Between Mobile Health App Engagement and Weight Loss and Glycemic Control in Adults With Type 2 Diabetes and Prediabetes (D’LITE Study): Prospective Cohort Study

**DOI:** 10.2196/35039

**Published:** 2022-09-30

**Authors:** Su Lin Lim, Melissa Hui Juan Tay, Kai Wen Ong, Jolyn Johal, Qai Ven Yap, Yiong Huak Chan, Genevieve Kai Ning Yeo, Chin Meng Khoo, Alison Yaxley

**Affiliations:** 1 Department of Dietetics National University Hospital Singapore Singapore; 2 Biostaistics Unit Yong Loo Lin School of Medicine National University Singapore Singapore Singapore; 3 Division of Endocrinology Department of Medicine National University Hospital Singapore Singapore; 4 Yong Loo Lin School of Medicine National University Singapore Singapore Singapore; 5 Nutrition and Dietetics, College of Nursing and Health Sciences Flinders University Adelaide Australia

**Keywords:** engagement, diabetes, prediabetes, mobile health, mHealth, mobile apps, weight loss, glycemic control, glycated hemoglobin, HbA1c change, mobile phone

## Abstract

**Background:**

Mobile health apps are increasingly used as early intervention to support behavior change for diabetes prevention and control, with the overarching goal of lowering the overall disease burden.

**Objective:**

This prospective cohort study conducted in Singapore aimed to investigate app engagement features and their association with weight loss and improved glycemic control among adults with diabetes and prediabetes from the intervention arm of the Diabetes Lifestyle Intervention using Technology Empowerment randomized controlled trial.

**Methods:**

Diabetes and prediabetes participants (N=171) with a median age of 52 years, BMI of 29.3 kg/m^2^, and glycated hemoglobin (HbA_1c_) level of 6.5% and who were being assigned the Nutritionist Buddy Diabetes app were included. Body weight and HbA_1c_ were measured at baseline, 3 months, and 6 months. A total of 476,300 data points on daily app engagement were tracked via the backend dashboard and developer’s report. The app engagement data were analyzed by quartiles and weekly means expressed in days per week. Linear mixed model analysis was used to determine the associations between the app engagements with percentage weight and HbA_1c_ change.

**Results:**

The median overall app engagement rate was maintained above 90% at 6 months. Participants who were actively engaged in ≥5 app features were associated with the greatest overall weight reduction of 10.6% from baseline (mean difference −6, 95% CI −8.9 to −3.2; *P*<.001) at 6 months. Adhering to the carbohydrate limit of >5.9 days per week and choosing healthier food options for >4.3 days per week had the most impact, eliciting weight loss of 9.1% (mean difference −5.2, 95% CI −8.2 to −2.2; *P*=.001) and 8.8% (mean difference −4.2, 95% CI −7.1 to −1.3; *P*=.005), respectively. Among the participants with diabetes, those who had a complete meal log for >5.1 days per week or kept within their carbohydrate limit for >5.9 days per week each achieved greater HbA_1c_ reductions of 1.2% (SD 1.3%; SD 1.5%), as compared with 0.2% (SD 1%; SD 0.6%). in the reference groups who used the features <1.1 or ≤2.5 days per week, respectively.

**Conclusions:**

Higher app engagement led to greater weight loss and HbA_1c_ reduction among adults with overweight or obesity with type 2 diabetes or prediabetes.

**Trial Registration:**

Australian New Zealand Clinical Trials Registry (ANZCTR) ACTRN12617001112358; https://anzctr.org.au/Trial/Registration/TrialReview.aspx?ACTRN=12617001112358

## Introduction

### Background

Globally, 374 million people are at an increased risk of developing type 2 diabetes [1]. With the increasingly urbanized and aging population, these numbers are expected to increase to 700 million by 2045 [1]. In Singapore, diabetes accounts for 8.6% of the total disease burden [2]. The prevalence of diabetes in Singapore increased from 8.2% in 2004 to 8.8% in 2017, with the latest prevalence at 9.5% in 2020 [3]. Of greater concern, 1 in 3 patients with diabetes has poor control of their condition and is at increased risk of a host of diabetes-related complications [4]. In addition, people with prediabetes who make up 14.4% of the Singapore population have a one-third chance of developing diabetes in the next 8 years [4]. Therefore, preventing the progression from prediabetes to diabetes and slowing the progression of diabetes are of utmost importance.

Weight reduction is associated with prevention and slowing of diabetes progression in patients with overweight or obesity with prediabetes or diabetes [5]. A 5% weight loss is associated with improved insulin sensitivity, better glycemic control, and reduced need for diabetes medications [5,6]. A 1% decrease in glycated hemoglobin (HbA_1c_) has been found to decrease death by 21%, myocardial infarction by 14%, and microvascular complications by 37% [7].

Apart from receiving medical care from health care providers, self-management (eg, monitoring of food intake, weight, and blood glucose) is an integral part of diabetes management to achieve sustainable health outcomes. In line with the self-regulation theory, patients with good self-management practices showed better management of their diabetes compared with patients who were simply prescribed medications [8]. In addition, good self-management practices can help patients to lose weight and improve hypertension and hyperlipidemia, which are key cardiovascular risk factors [9].

Numerous mobile apps have been developed to promote diabetes self-management. A meta-analysis with follow-up periods of approximately 6 months revealed a significant HbA_1c_ reduction (mean difference 0.49%, 95% CI 0.30-0.68) through diabetes self-management via mobile phone interactions [8]. Similarly, another meta-analysis of diabetes apps specifically designed to improve self-management practices reported a statistically significant reduction in body weight (mean difference 0.84 kg, 95% CI 0.17-1.51) among participants with diabetes [9].

There is limited research assessing users’ app engagement and the association with weight and HbA_1c_ changes in people with diabetes. App engagement studies were not focused on diabetes or had limited description on how engagement data were derived [10]. The question remains as to which app engagement features are associated with weight loss and improved glycemia to replicate similar findings in the real-world application. This prospective cohort study would add insight to the effectiveness of key app engagement functions associated with metabolic benefits among a group of individuals with diabetes risk and non–insulin-dependent diabetes.

### Objective

The primary Diabetes Lifestyle Intervention using Technology Empowerment (D’LITE) study has shown that both participants with prediabetes or diabetes achieved significant weight loss with a mean difference of −3.1 kg (95% CI −4.5 to −1.7; *P*<.001) and −2.4 kg (95% CI −3.5 to −1.3; *P*<.001) at 6 months, respectively, when compared with the control group [11,12]. The participants with prediabetes were 2.1 times likely to achieve normoglycemia (defined as HbA_1c_<5.7%) than in the control group (*P*<.018). Participants with diabetes also experienced a significant decrease in HbA_1c_ levels (mean change −0.7%, SD 1.2% vs −0.3%, SD 1.0%; *P*<.01) [11]. This further accentuates the need for investigating the various engagement levels within the app and its association with weight and HbA_1c_ reduction.

Therefore, the primary aim of our study was to investigate the association between participant engagement with a diabetes app and weight change and glycemic control in adults with diabetes and prediabetes. The findings of this study would provide insights on how diabetes apps could be used effectively to facilitate positive behavioral changes to improve health outcomes.

## Methods

### Study Design

This prospective cohort study included prespecified subgroup analysis of all participants from the intervention arm of the D’LITE study (N=171) who were assigned the Nutritionist Buddy Diabetes (nBuddy Diabetes) app [11,12]. A full description of the D’LITE study and the intervention details for both diabetes and prediabetes groups have previously been published [11,12].

The conceptualization of the nBuddy Diabetes app was based on behavioral science and the app was built with an extensive local food database and culturally appropriate automated cues [11]. In brief, the nBuddy Diabetes app comprises multiple features intended to support a participant’s self-management efforts including self-monitoring features of meal logging, calorie (CAL) and carbohydrate (CHO) limit alerts, and step tracking, which relies on the phone’s built-in pedometer and syncing with the user’s mobile phone. CAL and CHO limits were autocalculated by the app and individualized based on the participants’ input of their current weight, age, gender, and activity level in the app. When the CAL or CHO limit is reached per meal or per day, the automated cues designed with behavioral science embedded in the app algorithm will send a real-time prompt to remind participants to make a healthier meal choice. In addition, the app provides outcome tracking features such as weight charting and self-monitoring of blood glucose (SMBG), fasting and random blood glucose (RBG), to be inputted by the participants. A chat function involving 2-way communication between the dietitian and participants to facilitate individual lifestyle modification and coaching were made available within the app. Educational videos were uploaded onto the app and participants were notified upon upload via the chat function. Automated suggestions of healthier and culturally appropriate food alternatives and reminders for participants to engage with the app were also included.

At baseline, participants were taught to download and use the nBuddy Diabetes app to facilitate weight loss and glycemic control. Participants were advised to track their food and exercise daily while measuring their body weight twice weekly. During the first 3 months, participants were advised to measure their blood glucose 2 days per week. Participants were provided with a glucometer (FreeStyle Optium Neo) and a digital weighing scale (Omron HN-289). They were encouraged to achieve daily step count goals starting with an initial 3000 steps in the first week, 7000 in the second week, and 10,000 by the third week. Participants were advised to keep within the individualized CAL and CHO limits that were automatically calculated by the app based on the users’ profile.

### Setting and Participants

The study recruitment was conducted at government polyclinics, general practitioner clinics, health screening facilities, and hospital outpatient clinics in Singapore from October 2017 to September 2019. The inclusion criteria were adults who were 21 to 75 years of age, were literate in English, had a diagnosis of type 2 diabetes or prediabetes, had a BMI of ≥23 kg/m^2^, had a smartphone, and had provided written informed consent. Patients were excluded if they had been diagnosed with heart failure, advanced kidney disease, type 1 diabetes, severe cognitive or psychological disabilities, untreated hypothyroidism, thalassemia, or blood disorders or were pregnant. In addition, participants with insulin use, noncompliance to prescribed medications, and anemia were also excluded.

### Outcome Variables

Participants were assessed at baseline, 3 months, and 6 months from enrollment. The outcomes of interest were percentage changes in weight and HbA_1c_ levels from baseline to 3 months and 6 months. Body weight was measured using a calibrated digital weighing scale (Omron HN-289) at the clinic, while blood samples were obtained by a research assistant to determine HbA_1c_ levels following the standard methods of testing at National University Hospital Department of Laboratory Medicine and National Healthcare Group Diagnostics (both accredited by the College of American Pathologists). Reductions of 0.5% of HbA_1c_ levels and weight loss of ≥5% are considered clinically meaningful improvements associated with a decrease in cardiovascular risk in patients with diabetes in 12 months [13,14]. As such, the cut-offs of ≥5% weight loss and ≥0.5% HbA_1c_ reduction were chosen for use in interpretation of the data.

### Data Sources

App engagement data during the intervention period were tracked via the app’s backend dashboard and developer’s report. A total of 476,300 data points from the 171 participants were extracted. To coincide with the outcome measurements, the data were analyzed at 2 separate periods from baseline to 3 months and baseline to 6 months.

App engagement was defined as actively using the individual app features. For example, actively using the app features such as entering a body weight value was considered an app engagement while browsing or scrolling through the app was not. With the exception of videos watched, engagement data of all app features were tracked daily and the weekly mean days were derived for baseline to 3 months and baseline to 6 months. Videos watched, on the other hand, were calculated out of 22 videos that were uploaded via the app across 6 months. The exact definitions and derivations of the respective app engagements are presented in Table 1. The app engagement data were categorized into quartiles for comparison and analysis purposes.

**Table 1:** App engagement definitions.

App engagement	Definitions
Complete meal log	Considered complete if breakfast, lunch, and dinner were logged for the day. However, during Ramadan (Muslim fasting month), breakfast and dinner logged were considered the complete meal log for Muslim participants. The result is presented as the number of days participants had a complete meal log per week.
Any meal log (include incomplete meal log)	Number of days participants keyed in at least 1 food entry per week.
Within carbohydrate limit	Number of days participants kept within their carbohydrate limit as set by the app (only among the participants who had complete meal log) per week.
Within calorie limit	Number of days participants kept within their calorie limit as set by the app per week (only among the participants who had complete meal log).
Choosing healthier food options	Number of days participants consistently selected food choices labeled as healthier choices by the app per week.
Fasting blood glucose measurement	Number of days fasting blood glucose readings were recorded per week.
Random blood glucose measurement	Number of days random blood glucose readings were recorded per week.
Weight charting	Number of days weight was charted per week.
Achieving step count goal	Number of days participants achieved their step count goal per week.
Communication with dietitian	Number of days participants messaged the dietitian in the app per week.
Videos watched	Total number of videos watched during the 6 months.
Overall app use	Number of days participants actively use ≥1 features of the app per week.
App features with ≥75% uptake	App features (any meal log, within carbohydrate limit, within calorie limit, consistent healthier food choices, fasting blood glucose measurement, random blood glucose measurement, weight charting, achieving step count goal, communication with dietitian, and videos watched) with ≥75% uptake across 6 months.

The number of app engagement features with ≥75% uptake was also calculated. With 75% being considered a common and realistic uptake as reiterated by similar mobile health (mHealth) studies in the literature, it was used as a cut-off for more meaningful comparison of data with pre-existing literature [15]. On the basis of the cross-tabulation analysis, the results from 5 features and beyond rendered no additional effect and was taken as the minimal cut-off point for the test of significance.

### Statistical Methods

All analyses were performed using SPSS for Windows (version 26.0; SPSS Inc). Descriptive data for continuous variables were presented as median (IQR) or frequencies and percentages for categorical variables. Differences in continuous variables were assessed using the 2-sample *t* test when normality and homogeneity assumptions were satisfied; otherwise, the Mann-Whitney *U* test was used. The chi-square or Fisher exact test was used for categorical variables. The primary unit of analyses was the percentage change in weight and absolute HbA_1c_ levels at months 3 and 6 from baseline. Associations among app engagement behaviors, overall app use rate, and app features with ≥75% uptake on the outcomes were assessed using the Linear Mixed Model analysis to account for the clustering effect of recruitment sources as a random factor, adjusting for demographic and relevant covariates. Subgroup analyses of participants with diabetes and prediabetes were performed to investigate the associations. The app engagement data were categorized into quartiles for comparison and analysis purposes. The lowest quartile of app engagement was used as the reference category. Statistical significance was set at *P*<.05 (2-sided). Data were analyzed using the on-treatment approach, with missing data assumed as noncompliance to the intervention.

### Ethics Approval

The study was approved by the National Health care Group Domain Specific Review Board in Singapore (2017/00397), conducted in accordance with the Declaration of Helsinki and aligned with the Strengthening the Reporting of Observational Studies in Epidemiology guidelines [16].

## Results

### Participants’ Descriptive Data

Table 2 describes the baseline characteristics of the participants. A total of 171 participants were assigned to the mobile app group. Of the 171 participants, there were 99 (57.9%) participants with diabetes and 72 (42.1%) participants with prediabetes. At 6 months, 5 participants from the prediabetes group, 5 from the diabetes group, and an additional participant from the diabetes group who missed his 6-month outcome measurements were considered lost to follow up. Of 171, there were 109 (63.7%) males.

**Table 2:** Demographics of study participants at baseline (N=171).

Characteristics			All participants		Participants with DM^a^ (n=99)		Participants with PreDM^b^ (n=72)
Age (years), median (IQR)			52 (44-59)		52 (44-59)		52 (46-59)
**Sex, n (%)**							
	Male	109 (63.7)		66 (66.7)		43 (59.7)	
	Female	62 (36.3)		33 (33.3)		29 (40.3)	
**Ethnicity, n (%)**							
	Chinese	123 (71.9)		66 (66.7)		57 (79.2)	
	Malay	25 (14.6)		18 (18.2)		7 (9.7)	
	Indian	18 (10.5)		11 (11.1)		7 (9.7)	
	Others	5 (2.9)		4 (4)		1 (1.4)	
**Clinical variables, median (IQR)**							
	Weight (kg)	82.6 (74.2-90.3)		82.6 (75.6-90.8)		82.0 (73.0-89.4)	
	BMI (kg/m^2^)	29.3 (27.1-32.4)		29.8 (27.4-32.4)		28.9 (26.9-32.4)	
	HbA_1c_^c^ (%)	6.5 (5.9-7.5)		7.3 (6.6-8.0)		5.9 (5.7-6.2)	
	Fasting blood glucose (mmol/L)	6.8 (5.9-7.9)		7.8 (6.6-8.7)		6.0 (5.7-6.6)	
**Comorbidity, n (%)**							
	Hypertension	119 (69.6)		62 (62.6)		57 (79.2)	
	Hyperlipidemia	120 (70.2)		62 (62.6)		58 (80.6)	
Years of diagnosis, mean (SD)			N/A^d^		5.2 (4.1)		2.3 (2.5)

^a^DM: diabetes.

^b^PreDM: prediabetes.

^c^HbA_1c_: glycated hemoglobin.

^d^N/A: not applicable.

### Engagement Rates of nBuddy Diabetes App Features

The overall app use was high for the first 3 months and maintained throughout the 6 months (Table 3). Median overall app engagement rate remained high at above 90% over the course of the intervention. The most used features included step tracking (95.6%), meal logging (76.6%), and communication with the dietitian within the app’s chat system (50%). The least used features were RBG monitoring (18%), fasting blood glucose monitoring (19%), and weight charting (26%). This was anticipated owing to prior instructions given to the participants on the frequency (twice a week) of weight charting and SMBG. The trends in the app engagement were similar at baseline to 3 months and 6 months.

**Table 3:** Engagement rates of the nBuddy^a^ Diabetes app features (N=171).

App features engagement		Baseline to 3 months (%)	Baseline to 6 months (%)
**Overall app use**			
	Values, median (IQR)	97.8 (78.9-100.0)	91.7 (60.0-100.0)
	Value, range	8.9-100.0	9.4-100.0
**Meal logging**			
	Value, median (IQR)	76.6 (54.0-98.0)	71.0 (30.0-94.0)
	Value, range	10.0-100.0	6.0-100.0
**Step tracking**			
	Values, median (IQR)	95.6 (77.8-100.0)	90.0 (59.4-98.9)
	Value, range	14.4-100.0	8.3-100.0
**FBG^b^ monitoring**			
	Values, median (IQR)	19.0 (8.0-30.0)	12.0 (4.0-19.0)
	Value, range	0-86.0	0-69.0
**RBG^c^ monitoring**			
	Values, median (IQR)	18.0 (7.0-30.0)	11.0 (3.0-17.0)
	Value, range	0-96.0	0-79.0
**Weight charting**			
	Values, median (IQR)	26.0 (16.0-68.0)	18.0 (11.0-54.0)
	Value, range	3.0-97.0	2.0-98.0
**Communication with dietitian**			
	Values, median (IQR)	50.0 (29.0-67.0)	43.0 (23.0-63.0)
	Value, range	8.0-123.0	4.0-105.0
**Videos watched**			
	Values, median (IQR)	N/A^d^	32.0 (5.0-64.0)
	Value, range	N/A	0-100.0

^a^nBuddy: nutritionist buddy.

^b^FBG: fasting blood glucose.

^c^RBG: random blood glucose.

^d^N/A: not applicable.

### Associations Between App Engagement and Weight Change

**Figure 1:**
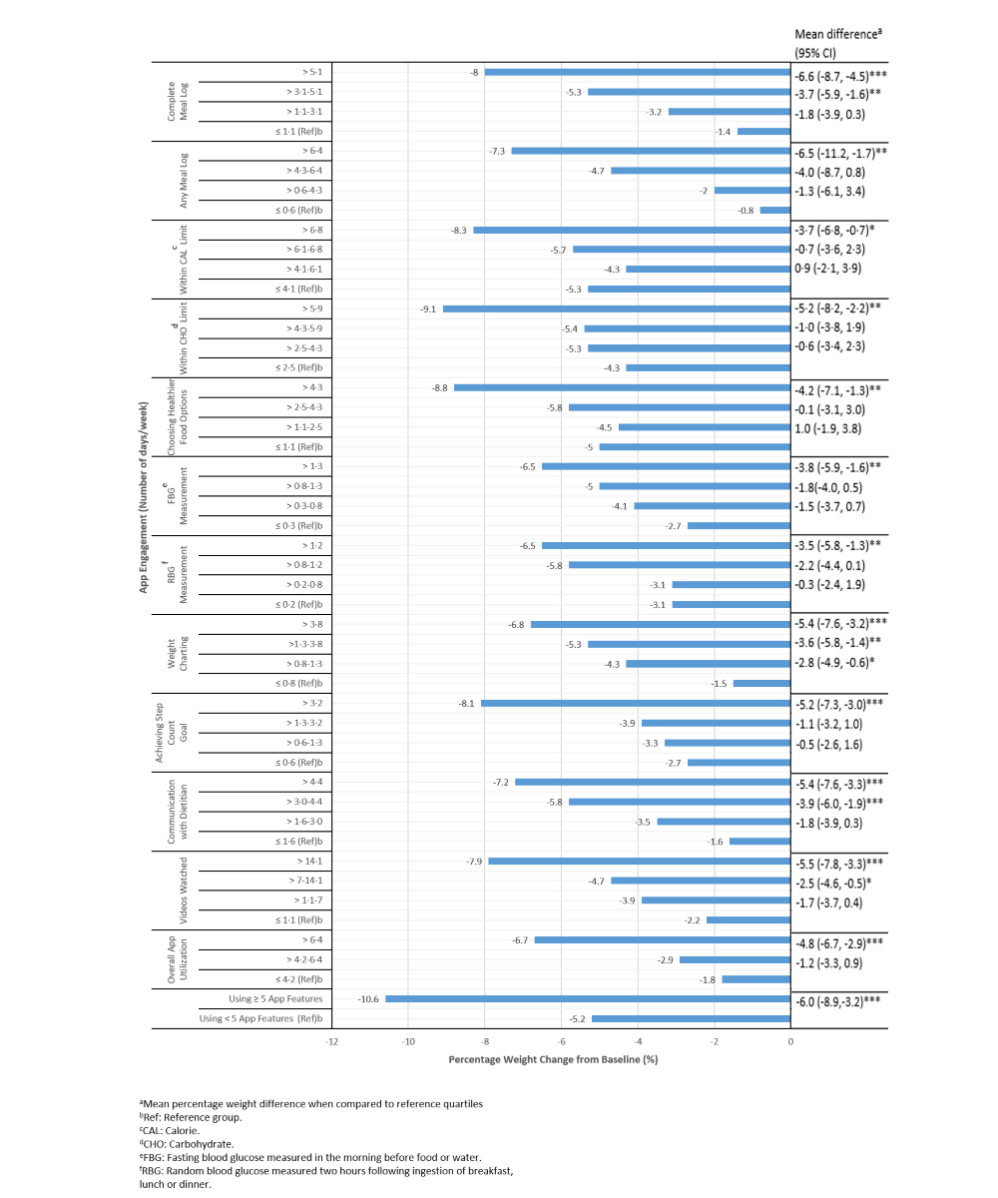
Association between app engagement and percentage weight change from baseline for all participants at 6 months (n=171). *P<.05. **P<.01. ***P<.001.

**Figure 2:**
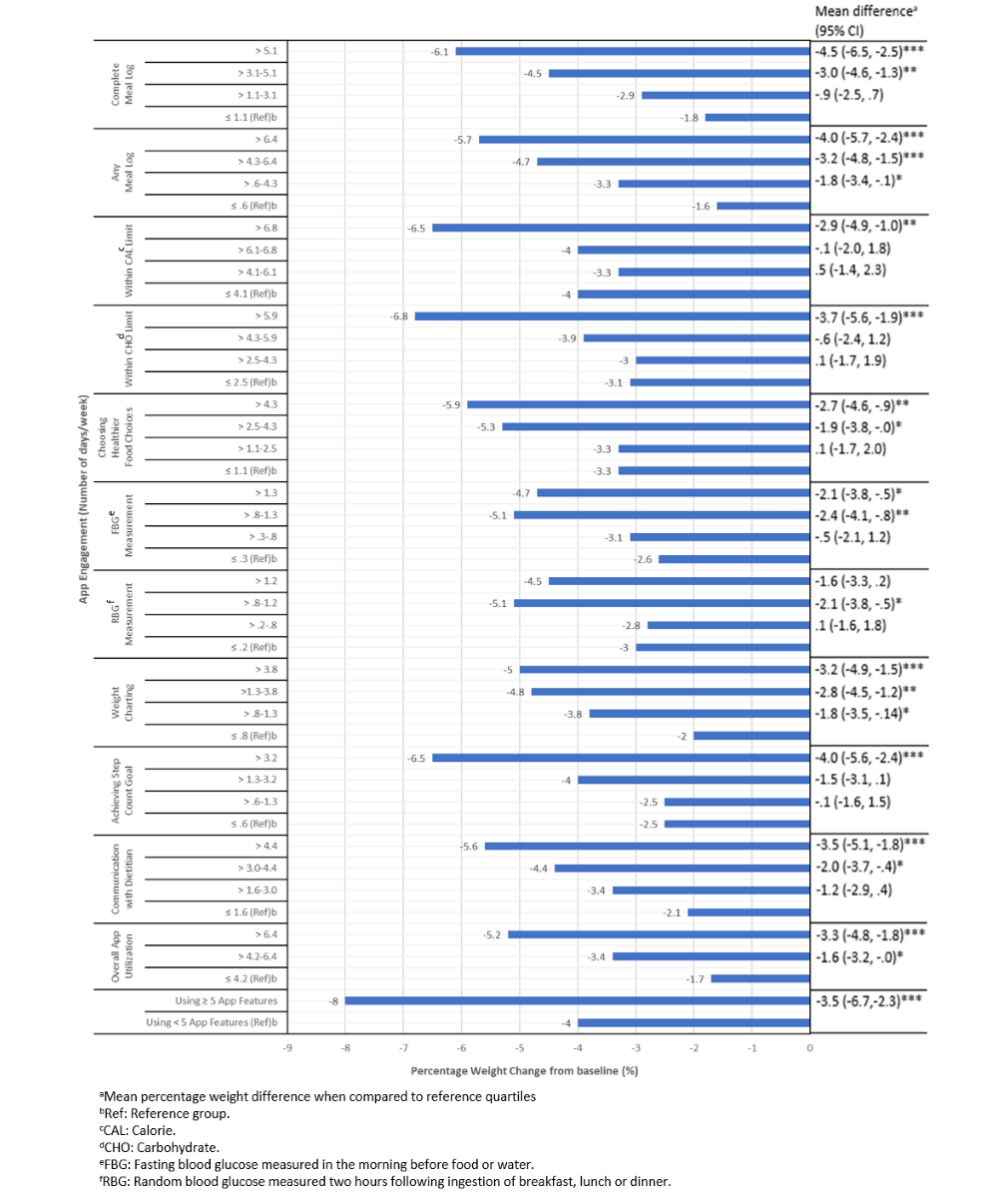
Association between app engagement and percentage weight change from baseline for all participants at 3 months (n=171). *P<.05. **P<.01. ***P<.001.

Figure 1 shows the association between app engagement and weight reduction among all participants at 6 months. The top quartiles of all app engagements achieved significantly greater weight loss at 6 months. The same weight loss trend was observed at 3 months, with the exception of RBG measurements (Figure 2). Among all the app features, the highest quartiles of within CHO limits and choosing healthier food options were associated with the greatest weight reductions of 9.1% and 8.8%, respectively (mean difference −5.2, 95% CI −8.2 to −2.2; *P*=.001; mean difference −4.2, 95% CI −7.1 to −1.3; *P*=.005; Figure 1). The overall use of the app for >6.4 days per week could elicit a weight loss of 6.7% (mean difference −4.8, 95% CI −6.7 to −2.9; *P*<.001), as compared with using the app for ≤4.2 days per week. Similarly, engaging in ≥5 app features with ≥75% uptake was significantly associated with a weight loss of 10.6% from baseline (mean difference −6; 95% CI −8.9 to −3.2; *P*<.001).

Among participants with prediabetes or diabetes, the highest quartiles of app engagement for all the app features led to ≥5% weight loss at 6 months (Multimedia Appendix 1). This trend was observed as early as 3 months whereby the highest quartile engagement levels of almost all the app features led to ≥5% weight loss (Multimedia Appendix 2). Complete meal log, keeping within CAL and CHO limits and choosing healthier food options, elicited the greatest weight loss of ≥8% when these app features were used most frequently (Multimedia Appendix 1).

In addition, overall use of the app for >6.4 days per week led to a greater weight loss of 6.8% compared with 2.1% weight loss for app use of ≤4.2 days per week (*P*=.009) among the prediabetes group at 6 months (Multimedia Appendix 1). A similar trend was observed in the diabetes group. Engaging in ≥5 app features with ≥75% uptake elicited an overall weight loss of 9.8% and 11.9% among the participants with diabetes and prediabetes, respectively.

Upon examining the app engagements efficiency, the app features that stood out for weight loss among the participants with prediabetes were complete meal log, within the CHO limit, RBG measurement, achieving step count goal, and communication with the dietitian (*P*<.05; Multimedia Appendix 1). Meanwhile, among the participants with diabetes, to attain both weight loss and HbA_1c_ reduction, the features that stood out were complete or any meal log, RBG measurement, weight charting, communication with dietitian, and videos watched (*P*<.01; Multimedia Appendices 1 and 3).

### Associations Between App Engagement and HbA_1c_ Change

Multimedia Appendices 3 and 4 illustrate that the higher the app engagement quartiles, the greater the HbA_1c_ reduction. As expected, HbA_1c_ reduction was more pronounced among participants with diabetes (*P*<.05) than participants with prediabetes among all app engagement. Among the participants with diabetes, all app engagements at the highest quartiles had clinically meaningful HbA_1c_ reduction of between 0.9% and 1.4% at 3 months and 6 months (*P*<.05 for all; Multimedia Appendices 3 and 4). Among the app features, meal logging, keeping within CAL and CHO limits, choosing healthier food options, fasting blood glucose and RBG measurements, weight charting, and achieving step count goal elicited the greatest impact on HbA_1c_ reduction of ≥1.2%, when used most frequently (Multimedia Appendix 3).

Participants with diabetes who had a complete meal log for >5.1 days per week or kept within their CHO limit of >5.9 days per week each achieved greater HbA_1c_ reductions of 1.2% (SD 1.5%) versus 0.2% (SD 0.6%) in those who logged their meals ≤1.1 days or kept within CHO ≤2.5 days per week (Multimedia Appendix 3). Overall app use of >6.4 days was associated with greater HbA_1c_ reduction (1.1% vs 0.3%) when compared with using the app for ≤4.2 days per week.

## Discussion

### Principal Findings

Our prospective study is significant in reporting the association between a diabetes app engagement and weight loss and HbA_1c_ change in adults with diabetes and prediabetes. Engaging with ≥5 app features with ≥75% uptake was associated with a substantial weight loss of 10.6% from baseline. Among participants with diabetes, greater app engagements led to higher improvement in glycemic control with HbA_1c_ reduction of between 1.0% and 1.4%. Our study results demonstrated that diabetes self-management through mobile phone app engagement was effective and sustainable at 6 months.

**Figure 3:**
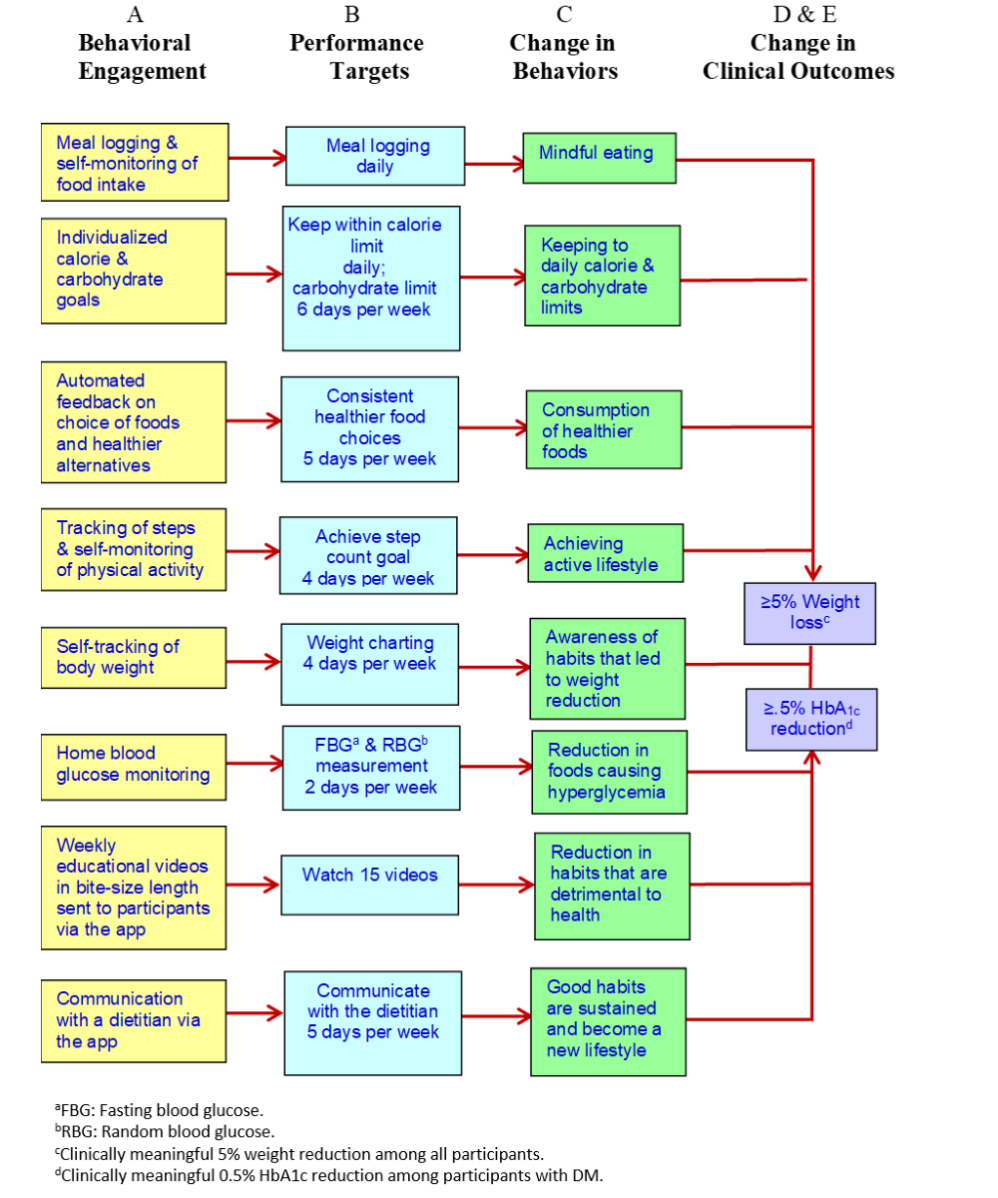
Effective behavioral treatment strategies in the nBuddy Diabetes App to optimize blood glucose control and weight loss (adapted from Lim et al [11]).

Past weight loss studies have reported better health outcomes with higher app engagement [17], emphasizing that higher app engagement is the primary determinant in successful weight loss [10]. Our findings are consistent with Painter et al [17] who found that a higher frequency of food log days, self-weighing entry days, or higher step counts per week was significantly associated with greater weight loss among participants with overweight and obesity. Our study revealed that the higher frequency of app use, the higher likelihood of achieving weight loss and HbA_1c_ reduction at 6 months*.* Moreover, the top quartiles of overall app use among all participants were significantly associated with a greater weight loss and HbA_1c_ reduction. We postulate that the more time spent on the app, the participants are more likely to engage with learning, self-monitoring, and health improvement behaviors that in turn lead to better self-management capability and commitment [18]. The self-regulation theory also states that self-monitoring and evaluation of one’s behaviors will lead to self-reinforcements, which in turn support behavior change toward attaining better health outcomes [17]. As the nBuddy Diabetes app was conceptualized based on a theoretical behavioral model [11], the results of this study provide evidence of the degree of app engagement for achieving clinically meaningful weight and HbA_1c_ reduction within 6 months. Figure 3 describes how our study findings align with the self-regulation theory to bring positive changes in behavior and health outcomes.

Meal logging has been identified as a commonly used feature among diabetes mHealth apps [19]. It is well-known that meal logging and tracking facilitate healthy dietary modifications [20]. Similarly, meal logging is one of the most used features in this study. Our study findings further strengthened the evidence of the link between meal logging via the app and improvements in weight and glycemic control [21]. Finally, Ingels et al [20] emphasized the importance of frequent and consistent dietary tracking for successful long-term weight loss. Taken together, meal logging should be made part of routine monitoring, similar to SMBG, not just to guide management for patients with diabetes during clinic visits but also as an important behavioral intervention.

It is also important to note that participants communicated with the dietitian through the app every other day. This feature provides an avenue for the user to clarify and ask questions pertaining to diabetes or weight control. It has been shown that SMBG with education and proper feedback improves diabetes control [22]. The 2-way communication with a dietitian could empower participants to make immediate changes based on the SMBG readings, meal log, and physical activity. Indeed, the engagement with the dietitian through the app was associated with a significant reduction in body weight and HbA_1c_ levels.

Apart from being one of the most used features, step counting was associated with clinically meaningful and statistically significant weight and HbA_1c_ reductions. Step counters with predetermined goals have been effective in forming good walking habits [5,23]. Our study findings agree with a meta-analysis that highlighted the benefits of pedometer use on weight loss among adults with overweight and obesity with diabetes [23]. Contrary to our findings, a meta-analysis and systematic review reported inconclusive glycemic effect and relationship around step count goals among patients with diabetes [24].

A study on diabetes apps found that inclusion of approximately 6 features led to both short- and long-term weight loss [25]. The use of a combination of features is also akin to the concepts of health care bundles consisting of 3 to 5 evidence-based practices to manage health care conditions [26]. Moreover, this study showed a greater HbA_1c_ reduction with ≥75% uptake of ≥5 app features at 3 and 6 months compared with using ≤5 features. In addition, our study emphasized complete meal log, within CHO limit, RBG measurement, achieving step count goal, and communication with dietitian for attaining weight loss among participants with prediabetes. On the other hand, complete meal log or any meal log, RBG measurement, weight charting, communication with the dietitian, and videos watched were crucial for participants with diabetes. Furthermore, Painter et al [17] highlighted the importance of self-monitoring features for better outcomes, and Van Rhoon et al [27] recommended a mixture of passive and interactive features. A recently published meta-analysis and systematic review echoed a similar conclusion as the authors concluded that the inclusion of an app to multicomponent usual care leads to greater weight loss [28].

The decline in app use over time was expected and has been commonly cited in mHealth app interventions [27]. However, the overall high app use was sustainable in our study at 3 months and 6 months. This could be attributed to the design features of the app, such as prompts that served as reminders for participants to use the app, and the chat function that was among the most used features in this study. The presence of a dietitian or health coach support could have assisted in optimal health information acquisition, learning, and application [29]. Moreover, the chat function has the potential to address patient lapses through reminders and deliver real-time tailored and dynamic behavioral interventions to support patient compliance with dietary and exercise recommendations [30]. This is also supported by past studies that reported significant associations between physician-patient communication and weight loss and HbA_1c_ reduction [29]. Several diabetes apps have not only echoed the importance of the 2-way chat communication but also highlighted its effectiveness in influencing behavior change [25].

### Strengths and Limitations

With the COVID-19 pandemic, the adoption of this locally contextualized app-based intervention, such as the nBuddy Diabetes app, could help practitioners facilitate better care and improve patients’ self-management in diabetes at the population level. The strengths of this study are the prospective tracking of health outcomes and the large number of app engagement data sets that enabled us to study the individual effect of specific app features on achieving desirable weight loss and blood glucose control. Similar to findings from the literature [28,31], our low attrition rate also illustrated that facilitating communication with a dietitian in the app could lead to greater and sustained engagement in diabetes self-management.

A limitation of this study is that participants who were willing to participate in this study displayed some degree of readiness for change with digital health literacy, and hence, the effect on unmotivated participants without digital health literacy remains unexamined. Participants may have also sought external input such as engaging in other health interventions or using other health apps, making it difficult to attribute success in weight loss and glycemic control solely to the app.

### Conclusions

In conclusion, engagement with the nBuddy Diabetes self-management app could elicit meaningful weight loss and HbA_1c_ reduction among individuals with overweight or obesity with prediabetes or diabetes. The greater the engagement with the app, the greater the weight loss and HbA_1c_ reduction.

## Appendix

Multimedia Appendix 1 Associations between app engagement and percentage weight change at 6 months for prediabetes or diabetes.

Multimedia Appendix 2 Associations between app engagement and percentage weight change at 3 months for prediabetes or diabetes.

Multimedia Appendix 3 Associations between app engagement and glycated hemoglobin change at 6 months for prediabetes or diabetes.

Multimedia Appendix 4 Associations between app engagement and glycated hemoglobin change at 3 months for prediabetes or diabetes.

## Abbreviations

CAL: calorie
CHO: carbohydrate
D’LITE: Diabetes Lifestyle Intervention Using Technology Empowerment
HbA_1c_: glycated hemoglobin
mHealth: mobile health
nBuddy: Nutritionist Buddy
RBG: random blood glucose
SMBG: self-monitoring of blood glucose
