# Supplementary material for: Association Between Mobile Health App Engagement and Weight Loss and Glycemic Control in Adults With Type 2 Diabetes and Prediabetes (D’LITE Study): Prospective Cohort Study
Source: JMIR Diabetes. 2022 Sep 30;7(3):e35039. doi: 10.2196/35039 (PMC9568822; doi:10.2196/35039)
Supplement: Multimedia Appendix 1 [file diabetes_v7i3e35039_app1.docx]

**Multimedia Appendix 1**

Associations between app engagement and percentage weight change at 6 months for prediabetes and diabetes.

| App engagement  (number of days per week) | | **Participants with prediabetes (n=67)** | | | | **Participants with diabetes (n=93)** | | | | | |  |
| --- | --- | --- | --- | --- | --- | --- | --- | --- | --- | --- | --- | --- |
|  |  | Values, n | Weight change from baseline (%),  mean (SD) | Mean difference  (95% CI) | *P*  value^a^ | Values, n | | Weight change from baseline (%),  mean (SD) | Mean difference  (95% CI) | *P*  value^a^ | |  |
| **Complete meal log** | |  |  |  | **.006^b^** |  | |  |  | **<.001^b^** | |  |
|  | >5.1 | 67 | ˗8.0 (5.5) | ˗6.5 (˗10.3 to ˗2.6) | **.002^b^** | | 93 | ˗8.0 (6.5) | ˗7.1 (˗9.8 – ˗4.3) | **<.001^b^** | | |
|  | >3.1 to 5.1 |  | ˗5.9 (4.3) | ˗2.6 (˗5.9 to 0.81) | .13 | |  | ˗4.7 (3.7) | ˗4.0 (˗7.0 – ˗1.1) | **.008^b^** | | |
|  | >1.1 to 3.1 |  | ˗3.0 (3.8) | 0.0 (˗3.3 to 3.4) | .98 | |  | ˗3.4 (4.6) | ˗2.0 (˗4.8 – 0.8) | .16 | | |
|  | ≤1.1 (Ref^c^) |  | ˗2.3 (3.4) | — |  | |  | ˗0.9 (2.4) | — |  | | |
| **Any meal log** | |  |  |  | .06 |  | |  |  | **<.001^b^** | |  |
|  | >6.4 | 67 | ˗7.1 (5.3) | ˗9.3 (˗18.4 to ˗0.2) | **.046^b^** | | 93 | ˗7.4 (6.4) | ˗5.8 (˗11.5 – ˗0.1) | **.045 ^b^** | | |
|  | >4.3 to 6.4 |  | ˗4.9 (4.5) | ˗7.3 (˗16.4 to 1·9) | .12 | |  | ˗4.5 (4.8) | ˗3.2 (˗8.9 – 2.5) | .27 | | |
|  | >0.6 to 4.3 |  | ˗2.6 (2.9) | ˗5.7 (˗14.8 to 3.4) | .21 | |  | ˗1.8 (3.2) | ˗0.1 (˗5.0 – 5.6) | .97 | | |
|  | ≤0.6 (Ref^c^) |  | 2.4 (0) | — |  | |  | ˗1.8 (1.2) | — |  | | |
| **Within CAL^d^ limit** | | |  |  | .27 | |  |  |  | .08 | | |
|  | >6.8 | 45 | ˗8.5 (5.3) | ˗3.3 (˗8.3 to 1.8) | .20 | | 55 | ˗8.2 (6.8) | ˗3.5 (˗7.9 – 0.8) | .11 | | |
|  | >6.1 to 6.8 |  | ˗5.5 (6.7) | 0.3 (˗4.9 to 5.4) | .92 | |  | ˗5.9 (4.2) | 0.2 (˗4.1 – 4.4) | .94 | | |
|  | >4.1 to 6.1 |  | ˗5.1 (4.3) | 1.2 (˗3.0 to 5.5) | .57 | |  | ˗3.7 (5.0) | 2.3 (˗2.0 – 6.7) | .28 | | |
|  | ≤4.1 (Ref^c^) |  | ˗5.1 (4.4) | — |  | |  | ˗5.5 (5.1) | — |  | | |
| **Within CHO^e^ limit** | | |  |  | **.01^b^** |  | |  |  | .17 | |  |
|  | >5.9 | 46 | ˗10.9 (5.5) | ˗5.6 (˗9.8 to ˗1.3) | **.01^b^** | | 55 | ˗7.9 (7.0) | ˗4.8 (˗9.4 – ˗0.3) | **.04^b^** | | |
|  | >4.3 to 5.9 |  | ˗4.3 (4.9) | 1.1 (˗2.8 to 5.1) | .57 | |  | ˗6.1 (4.0) | ˗1.7 (˗6.2 – 2.7) | .44 | | |
|  | >2.5 to 4.3 |  | ˗4.8 (4.1) | 0.9 (˗2.6 to 4.4) | .61 | |  | ˗5.8 (4.8) | ˗1.2 (˗5.8 – 3.4) | .60 | | |
|  | ≤2.5 (Ref^c^) |  | ˗5.0 (4.2) | — |  | |  | ˗3.3 (5.4) | — |  | | |
| **Choosing healthier**  **food options** | | | |  | .08 |  | |  |  | .09 | |  |
|  | >4.3 | 46 | ˗9.4 (6.4) | ˗4.2 (˗8.5 to 0.2) | .06 | | 54 | ˗8.3 (6.4) | ˗4.5 (˗8.7 – ˗0.2) | **.04^b^** | | |
|  | >2.5 to 4.3 |  | ˗5.1 (4.0) | ˗0.5 (˗5.0 to 4.1) | .84 | |  | ˗6.3 (5.1) | 0.2 (˗4.2 – 4.6) | .92 | | |
|  | >1.1 to 2.5 |  | ˗4.1 (2.9) | 0.9 (˗3.3 to 5.1) | .68 | |  | ˗4.9 (5.7) | 0.7 (˗3.4 – 4.8) | .72 | | |
|  | ≤1.1 (Ref^c^) |  | ˗5.3 (5.5) | — |  | |  | ˗4.8 (4.5) | — |  | | |
| **FBG^f^ measurement** | |  |  |  | .34 |  | |  |  | **.02^b^** | |  |
|  | >1.3 | 67 | ˗6.5 (5.0) | ˗2.5 (˗6.0 to 1.0) | .16 | | 93 | ˗6.6 (5.6) | ˗4.6 (˗7.9 – ˗1.3) | **.006^b^** | | |
|  | >0.8 to 1.3 |  | ˗6.7 (5.0) | ˗1.9 (˗5.3 to 1.5) | .27 | |  | ˗4.3 (5.3) | ˗1.8 (˗5.2 – 1.6) | .29 | | |
|  | >0.3 to 0.8 |  | ˗6.0 (5.7) | ˗2.3 (˗5.4 to 0.7) | .13 | |  | ˗2.6 (4.8) | ˗0.8 (˗4.2 – 2.7) | .66 | | |
|  | ≤0.3 (Ref^c^) |  | ˗3.1 (3.4) | — |  | |  | ˗1.8 (2.7) | — |  | | |
| **RBG^g^ measurement** | |  |  |  | **.03^b^** |  | |  |  | **.04^b^** | |  |
|  | >1.2 | 67 | ˗7.7 (4.8) | ˗4.9 (˗8.2 to ˗1.5) | **.005^b^** | | 93 | ˗6.0 (4.8) | ˗3.3 (˗6.9 – 0.2) | .07 | | |
|  | >0.8 to 1.2 |  | ˗7.3 (5.3) | ˗2.7 (˗5.8 to 0.4) | .09 | |  | ˗4.8 (5.9) | ˗1.2 (˗4.8 – 2.5) | .53 | | |
|  | >0.2 to 0.8 |  | ˗4.1 (3.5) | ˗0.7 (˗3.6 to 2.2) | .65 | |  | ˗2.5 (4.6) | 0.5 (˗3.0 – 3.9) | .79 | | |
|  | ≤0.2 (Ref^c^) |  | ˗3.3 (4.5) | — |  | |  | ˗2.6 (5.0) | — |  | | |
| **Weight charting** | |  |  |  | .05 |  | |  |  | **.002^b^** | |  |
|  | >3.8 | 67 | ˗6.6 (4.9) | ˗4.8 (˗8.3 to ˗1.2) | **.01^b^** | | 93 | ˗7.2 (7.0) | ˗6.2 (˗9.4 – ˗3.1) | **<.001^b^** | | |
|  | >1.3 to 3.8 |  | ˗6.7 (5.8) | ˗4.6 (˗8.4 to ˗0.8) | **.02^b^** | |  | ˗4.5 (4.8) | ˗2.8 (˗5.5 – 0.0) | .05 | | |
|  | >0.8 to 1.3 |  | ˗3.9 (3.0) | ˗2.6 (˗6.3 to 1.0) | .16 | |  | ˗4.6 (5.4) | ˗3.2 (˗6.0 – ˗0.5) | **.02^b^** | | |
|  | ≤0.8 (Ref^c^) |  | ˗1.0 (2.0) | — |  | |  | ˗1.8 (2.8) | — |  | | |
| **Achieving step count goal** | |  |  |  | **<.001^b^** |  | |  |  | **.003^b^** | |  |
|  | >3.2 | 67 | ˗8.8 (4.9) | ˗6.9 (˗9.9 to ˗3.8) | **<.001^b^** | | 93 | ˗7.7 (6.4) | ˗4.8 (˗7.6 – ˗1.9) | **.001^b^** | | |
|  | >1.3 to 3.2 |  | ˗4.8 (5.2) | ˗2.0 (˗4.9 to 0.9) | .17 | |  | ˗3.3 (4.2) | ˗0.6 (˗3.4 – 2.2) | .66 | | |
|  | >0.6 to 1.3 |  | ˗3.9 (2.7) | ˗2.9 (˗5.8 to ˗0.01) | .05 | |  | ˗2.7 (4.3) | ˗0.4 (˗3.5 – 2.6) | .78 | | |
|  | ≤0.6 (Ref^c^) |  | ˗3.0 (4.6) | — |  | |  | ˗2.5 (3.6) | — |  | | |
| **Communication with dietitian** | |  |  |  | **.04^b^** |  | |  |  | **.001^b^** | |  |
|  | >4.4 | 67 | ˗7.4 (5.0) | ˗5.2 (˗8.6 to ˗1.7) | **.004^b^** | | 93 | ˗6.9 (6.2) | ˗5.2 (˗8.1 – ˗2.4) | **<.001^b^** | | |
|  | >3.0 to 4.4 |  | ˗5.3 (4.6) | ˗3.4 (˗6.8 – ˗0.03) | **.048^b^** | |  | ˗6.3 (5.4) | ˗4.4 (˗7.3 – ˗1.6) | **.003^b^** | | |
|  | >1.6 to 3.0 |  | ˗4.3 (4.6) | ˗3.1 (˗6.6 – 0.4) | .08 | |  | ˗2.8 (4.0) | ˗1.3 (˗4.0 – 1.4) | .35 | | |
|  | ≤1.6 (Ref^c^) |  | ˗1.6 (3.0) | — |  | |  | ˗1.6 (3.3) | — |  | | |
| **Videos watched** | |  |  |  | .39 |  | |  |  | **<.001^b^** | |  |
|  | >14.1 | 67 | ˗7.8 (6.0) | ˗3.2 (˗7.1 – 0.7) | .10 | | 93 | ˗7.9 (6.8) | ˗6.3 (˗9.2 – ˗3.4) | **<.001^b^** | | |
|  | >7.0 to 14.1 |  | ˗5.3 (4.9) | ˗0.9 (˗4.5 – 2.6) | .60 | |  | ˗4.2 (4.2) | ˗2.4 (˗5.1 – 0.4) | .09 | | |
|  | >1.1 to 7.0 |  | ˗4.7 (4.2) | ˗1.5 (˗4.7 – 1.7) | .36 | |  | ˗3.3 (4.6) | ˗1.7 (˗4.5 – 1.1) | .24 | | |
|  | ≤1.1 (Ref^c^) |  | ˗2.7 (3.1) | — |  | |  | ˗1.9 (3.3) | — |  | | |
| **Overall app utilization** | |  |  |  | **.02^b^** |  | |  |  | **<.001^b^** | |  |
|  | >6.4 | 67 | ˗6.8 (5.1) | ˗4.1 (˗7.1 – ˗1.1) | **.009^b^** | | 93 | ˗6.6 (6.2) | ˗5.1 (˗7.8 – ˗2.5) | **<·001^b^** |  |  |
|  | >4.2 to 6.4 |  | ˗3.6 (3.8) | ˗1.0 (˗4.3 – 2.2) | .52 | |  | ˗2.6 (3.0) | ˗1.3 (˗4.0 – 1.4) | .35 |  |  |
|  | ≤4.2 (Ref^c^) |  | ˗2.1 (3.0) | — |  | |  | ˗1.6 (2.7) | — |  |  |  |
| **Number of app features with ≥75% uptake** | | |  |  | **.003^b^** |  | |  |  | **.003^b^** | |  |
| ≥5  <5 | | 45 | ˗11.9 (5.6)  ˗5.0 (4.5) | ˗6.8 (˗11.2– ˗2.4)  — | **.003^b^** | 54 | | ˗9.8 (7.8)  ˗5.3 (4.7) | ˗6.1 (˗10.0 – ˗2.2)  — | **.003^b^** | |  |

^a^Adjusted for age, gender, and ethnicity.

^b^Statistically significant *P* values when compared with reference quartiles.

^c^Ref: reference group.

^d^CAL: calorie.

^e^CHO: carbohydrate.

^f^FBG: fasting blood glucose; measured in the morning before food or water.

^g^RBG: random blood glucose; measured 2 hours following ingestion of breakfast, lunch, or dinner.

— N/A for the reference groups.

This is a Multimedia Appendix to a full manuscript published in the J Med Internet Res Diabetes.
