# Supplementary material for: Association Between Mobile Health App Engagement and Weight Loss and Glycemic Control in Adults With Type 2 Diabetes and Prediabetes (D’LITE Study): Prospective Cohort Study
Source: JMIR Diabetes. 2022 Sep 30;7(3):e35039. doi: 10.2196/35039 (PMC9568822; doi:10.2196/35039)
Supplement: Multimedia Appendix 2 [file diabetes_v7i3e35039_app2.docx]

**Multimedia Appendix 2**

Associations between app engagement and percentage weight change at 3 months for prediabetes and diabetes.

| App engagement  (number of days/week) | | **Participants with prediabetes** | | | | | **Participants with diabetes** | | | | | | |
| --- | --- | --- | --- | --- | --- | --- | --- | --- | --- | --- | --- | --- | --- |
|  |  | Values, n | Weight change from baseline (%),  mean (SD) | Mean difference  (95% CI) | *P* value^a^ | | Values, n | | Weight change from baseline (%),  mean (SD) | Mean difference (95% CI) | | | *P* value^a^ |
| **Complete meal log** | |  |  |  | **.04^b^** | |  | |  |  | | | **.002^b^** |
|  | >5.9 | 69 | ˗5.7 (4.3) | ˗4.2 (˗8.1 – ˗0.3) | **.04^b^** | | 95 | | ˗6.4 (5.0) | ˗5.0 (˗7.6 – ˗2.5) | | | **<.001^b^** |
|  | >4.1 to 5.9 |  | ˗5.6 (3.4) | ˗2.8 (˗5.8 – 0.1) | .06 | |  |  | ˗3.8 (3.5) | ˗2.4 (˗4.7 – ˗0.2) | | | **.04^b^** |
|  | >1.9 to 4.1 |  | ˗2.8 (3.2) | 0.2 (˗2.5 – 2.8) | .91 | |  |  | ˗3.0 (2.9) | ˗1.7 (˗3.9 – 0.5) | | | .12 |
|  | ≤ 1.9 (Ref^c^) |  | ˗3.0 (3.4) |  |  | |  |  | ˗1.1 (1.9) |  | | |  |
| **Any meal log** | |  |  |  | **.02^b^** | |  | |  |  | | | **.004^b^** |
|  | >6.9 | 69 | ˗6.0 (3.5) | ˗4.5 (˗7.4 – ˗1.7) | **.003^b^** | | 95 | | ˗5.5 (4.5) | ˗3.7 (˗5.9 – ˗1.6) | | | **.001^b^** |
|  | >6.3 to 6.9 |  | ˗4.5 (4.4) | ˗2.8 (˗5.5 – ˗0.0) | **.047^b^** | |  |  | ˗4.9 (5.1) | ˗3.5 (˗5.7 – ˗1.2) | | | **.003^b^** |
|  | >3.8 to 6.3 |  | ˗3.7 (3.1) | ˗2.0 (˗4.9 – 0.8) | .16 | |  |  | ˗3.0 (3.2) | ˗1.5 (˗3.6 – 0.6) | | | .15 |
|  | ≤3.8 (Ref^c^) |  | ˗1.6 (2.1) |  |  | |  |  | ˗1.6 (1.9) |  | | |  |
| **Within CAL^d^ limit** | |  |  |  | .05 | |  | |  |  | | | **.06** |
|  | >6.7 | 56 | ˗7.4 (4.1) | ˗3.3 (˗6.4 – ˗0.2) | **.04^b^** | | 77 | | ˗6.0 (4.8) | ˗2.5 (˗5.2 – 0.2) | | | .07 |
|  | >6.0 to 6.7 |  | ˗3.9 (3.0) | 0.2 (˗2.7 – 3.0) | .91 | |  |  | ˗4.0 (4.6) | ˗0.1 (˗2.8 – 2.6) | | | .93 |
|  | >4.0 to 6.0 |  | ˗3.8 (3.7) | 0.8 (˗2.1 – 3.8) | .58 | |  |  | ˗2.9 (3.3) | 1.0 (˗1.6 – 3.6) | | | .44 |
|  | ≤4.0 (Ref^c^) |  | ˗4.2 (3.4) |  |  | |  |  | ˗3.8 (3.4) |  | | |  |
| **Within CHO^e^ limit** | |  |  |  | **.02^b^** | |  | |  |  | | | **.01^b^** |
|  | >5.7 | 58 | ˗7.4 (3.9) | ˗3.6 (˗6.5 – ˗0.7) | **.02^b^** | | 80 | | ˗6.4 (4.8) | ˗3.7 (˗6.2 – ˗1.2) | | | **.004^b^** |
|  | >3.8 to 5.7 |  | ˗4.2 (3.5) | ˗0.7 (˗3.5 – 2.0) | .59 | |  |  | ˗3.7 (3.5) | ˗0.5 (˗3.0 – 2.0) | | | .71 |
|  | >2.2 to 3.8 |  | ˗3.1 (3.2) | 0.8 (˗1.9 – 3.6) | .55 | |  |  | ˗3.0 (3.6) | 0.1 (˗2.4 – 2.6) | | | .96 |
|  | ≤2.2 (Ref^c^) |  | ˗3.8 (3.5) |  |  | |  |  | ˗2.7 (3.5) |  | | |  |
| **Choosing healthier food options** | | |  |  | .16 | |  | |  |  | | | **.04^b^** |
|  | >4.2 | 56 | ˗6.3 (4.5) | ˗2.5 (˗5.5 – 0.6) | .11 | | 77 | | ˗5.6 (5.7) | ˗3.3 (˗5.8 – ˗0.8) | | | **.01^b^** |
|  | >2.3 to 4.2 |  | ˗5.1 (3.8) | ˗1.4 (˗4.3 – 1.4) | .32 | |  |  | ˗5.4 (4.1) | ˗1.8 (˗4.4 – 0.8) | | | .17 |
|  | >0.9 to 2.3 |  | ˗3.5 (3.0) | 0.5 (˗2.5 – 3.5) | .73 | |  |  | ˗3.2 (2.6) | ˗0.2 (˗2.8 – 2.4) | | | .88 |
|  | ≤0.9 (Ref^c^) |  | ˗3.8 (3.0) |  | |  |  |  | ˗3.0 (3.3) |  | | |  |
| **FBG^f^ measurement** | |  |  |  | **<.001^b^** | |  | |  |  | | | **.01^b^** |
|  | >2.1 | 69 | ˗1.0 (2.1) | 2.5 (˗1.3 – 6.2) | .20 | | 95 | | ˗5.1 (4.2) | ˗2.9 (˗5.3 – ˗0.5) | | | **.02^b^** |
|  | >1.3 to 2.1 |  | ˗6.8 (3.8) | ˗4.1 (˗6.2 – ˗1.9) | **<.001^b^** | |  |  | ˗3.8 (4.4) | ˗1.4 (˗4.0 – 1.2) | | | .28 |
|  | >0.5 to 1.3 |  | ˗4.7 (3.6) | ˗1.7 (˗3.8 – 0.4) | .12 | |  |  | ˗1.8 (3.2) | 0.6 (˗2.1 – 3.2) | | | .88 |
|  | ≤0.5 (Ref^c^) |  | ˗2.9 (3.1) |  |  | |  |  | ˗2.0 (2.0) |  | | |  |
| **RBG^g^ measurement** | |  |  |  | .22 | |  | |  |  | | | .06 |
|  | >2.1 | 69 | ˗3.9 (4.4) | ˗0.6 (˗4.1 – 3.0) | .75 | | 95 | | ˗4.6 (4.1) | ˗2.1 (˗4.8 – 0.6) | | | .13 |
|  | >1.3 to 2.1 |  | ˗5.8 (4.1) | ˗2.4 (˗4.7 – ˗0.1) | **.04 ^b^** | |  |  | ˗4.6 (4.4) | ˗1.7 (˗4.4 – 1.1) | | | .22 |
|  | >0.5 to 1.3 |  | ˗4.2 (2.7) | ˗0.9 (˗3.4 – 1.7) | .49 | |  |  | ˗2.0 (3.3) | 0.5 (˗2.2 – 3.3) | | | .70 |
|  | ≤0.5 (Ref^c^) |  | ˗3.3 (3.6) |  |  | |  |  | ˗2.4 (3.4) |  | | |  |
| **Weight charting** | |  |  |  | .088 | |  | |  |  | | | **.02^b^** |
|  | >4.8 | 69 | ˗4.8 (3.7) | ˗2.4 (˗5.3 – 0.5) | .10 | | 95 | | ˗5.2 (5.0) | ˗3.5 (˗5.7 – ˗1.3) | | | **.002^b^** |
|  | >1.8 to 4.8 |  | ˗5.7 (4.5) | ˗3.6 (˗6.6 – ˗0.5) | **.02^b^** | |  |  | ˗4.1 (4.1) | ˗2.3 (˗4.4 – ˗0.2) | | | **.02^b^** |
|  | >1.1 to 1.8 |  | ˗3.3 (3.2) | ˗1.1 (˗4.0 – 1.9) | .48 | |  |  | ˗4.2 (4.2) | ˗2.4 (˗4.6 – ˗0.2) | | | **.03^b^** |
|  | ≤1.1 (Ref^c^) |  | ˗2.3 (2.2) |  |  | |  |  | ˗1.9 (2.3) |  | | |  |
| **Achieving step count goal** | |  |  |  | **.004^b^** | |  | |  |  | | | **<.001^b^** |
|  | >3.4 | 69 | ˗6.8 (4.1) | ˗4.7 (˗7.2 – ˗2.2) | **<.001^b^** | | 95 | | ˗6.3 (4.8) | ˗3.5 (˗5.6 – ˗1.4) | | | **.002^b^** |
|  | >1.9 to 3.4 |  | ˗4.0 (4.0) | ˗1.4 (˗3.9 – 1.0) | .24 | |  |  | ˗4.0 (3.6) | ˗1.1 (˗3.3 – 1.1) | | | .32 |
|  | >0.9 to 1.9 |  | ˗3.7 (3.1) | ˗1.3 (˗3.6 – 1.1) | .30 | |  |  | ˗1.5 (2.5) | 0.8 (˗1.4 – 2.9) | | | .48 |
|  | ≤0.9 (Ref^c^) |  | ˗2.5 (2.6) |  |  | |  |  | ˗2.4 (3.0) |  | | |  |
| **Communication with dietitian** | | |  |  | **.02^b^** | |  | |  |  | | | .107 |
|  | >4.7 | 69 | ˗6.1 (3.9) | ˗4.2 (˗6.8 – ˗1.6) | **.002^b^** | | 95 | | ˗5.0 (5.3) | ˗2.6 (˗4.9 – ˗0.3) | | | **.03^b^** |
|  | >3.5 to 4.7 |  | ˗4.0 (3.3) | ˗2.2 (˗4.7 – 0.4) | .10 | |  |  | ˗4.7 (3.3) | ˗1.9 (˗4.1 – 0.4) | | | .11 |
|  | >2.0 to 3.5 |  | ˗4.2 (3.7) | ˗2.4 (˗5.2 – 0.4) | .09 | |  |  | ˗2.9 (3.9) | ˗0.5 (˗2.6 – 1.7) | | | .66 |
|  | ≤2.0 (Ref^c^) |  | ˗1.8 (2.8) |  |  | |  |  | ˗2.3 (3.0) |  | | |  |
| **Overall app utilisation** | |  |  |  | **.004^b^** | |  | |  |  | | | **.03^b^** |
|  | >6.8 | 69 | ˗5.7 (4.1) | ˗3.8 (˗6.2 – ˗1.3) | **.003^b^** | | 95 | | ˗4.7 (4.7) | ˗2.8 (˗4.8 – ˗0.8) | | | **.007^b^** |
|  | >5.5 to 6.8 |  | ˗3.2 (2.9) | ˗1.1 (˗3.7 – 1.5) | .39 | |  |  | ˗3.6 (3.5) | ˗1.7 (˗3.9 – 0.4) | | | .12 |
|  | ≤5.5 (Ref^c^) |  | ˗2.0 (2.2) |  |  | |  |  | ˗1.6 (2.0) |  | | |  |
| **Number of app features with ≥75% uptake** | | |  |  | **.009^b^** | |  | |  |  | | | **.002^b^** |
| ≥5  <5 | | 56 | ˗8.2 (3.4)  ˗4.1 (3.5) | ˗4.0 (˗7.0 – ˗1.0) | **.009^b^** | | 77 | | ˗7.7 (5.9)  ˗3.9 (3.9) | ˗3.8 (˗7.3 – ˗0.4) | | | **.002^b^** |
|  |  |  |  |  |  | |  |  | |  |  |  |  |

^a^Adjusted for age, gender and ethnicity.

^b^Statistically significant *P* values when compared to reference quartiles.

^c^Ref: Reference group.

^d^CAL: Calorie.

^e^CHO: Carbohydrate.

^f^FBG: Fasting blood glucose measured in the morning before food or water.

^g^RBG: Random blood glucose measured two hours following ingestion of breakfast, lunch or

dinner.
