# Supplementary material for: Association Between Mobile Health App Engagement and Weight Loss and Glycemic Control in Adults With Type 2 Diabetes and Prediabetes (D’LITE Study): Prospective Cohort Study
Source: JMIR Diabetes. 2022 Sep 30;7(3):e35039. doi: 10.2196/35039 (PMC9568822; doi:10.2196/35039)
Supplement: Multimedia Appendix 3 [file diabetes_v7i3e35039_app3.docx]

**Multimedia Appendix 3**

Associations between app engagement and HbA_1c_^a^ change at 6 months for prediabetes and diabetes.

| App engagement  (number of days per week) | | **Participants with prediabetes (n=67)** | | | | | |  | | | **Participants with diabetes**  **(n=93)** | | | |  |
| --- | --- | --- | --- | --- | --- | --- | --- | --- | --- | --- | --- | --- | --- | --- | --- |
|  |  | Values, n | HbA_1c_  change  from  baseline  (%), mean  (SD) | | Mean difference  (95% CI) | | *P* value^b^ | Values, n | | HbA_1c_ change from baseline (%), mean (SD) | | Mean difference  (95% CI) | | *P*  value^b^ |  |
| **Complete meal log** | |  |  | |  | | **.008**^c^ |  | |  | |  | | **<.001**^c^ |  |
|  | >5.1 | 67 | ˗0.3 (0.3) | ˗0.4 (˗0.6 – ˗0.1) | | **.002**^c^ | | 93 | ˗1.2 (1.3) | | | ˗1.1 (˗1.6 – ˗0.7) | **<.001**^c^ | | |
|  | >3.1 to 5.1 |  | ˗0.3 (0.3) | ˗0.2 (˗0.4 – 0.0) | | .10 | |  | ˗0.8 (1.2) | | | ˗0.7 (˗1.2 – ˗0.3) | **.004**^c^ | | |
|  | >1.1 to 3.1 |  | ˗0.1 (0.2) | 0.0 (˗0.2 – 0.2) | | .93 | |  | ˗0.6 (1.0) | | | ˗0.5 (˗1.0 – ˗0.1) | **.03**^c^ | | |
|  | ≤1.1 (Ref ^d^) |  | ˗0.1 (0.3) | — | |  | |  | ˗0.2 (1.0) | | | — |  | | |
| **Any meal log** | |  |  | |  | | .26 |  | |  | |  | | **<.001**^c^ |  |
|  | >6.4 | 67 | ˗0.3 (0.3) | ˗0.3 (˗0.9 – 0.3) | | .27 | | 93 | ˗1.3 (1.4) | | | ˗1.4 (˗2.3 – ˗0.5) | **.004**^c^ | | |
|  | >4.3 to 6.4 |  | ˗0.2 (0.3) | ˗0.2 (˗0.8 – 0.4) | | .45 | |  | ˗0.7 (1.0) | | | ˗0.9 (˗1.9 – 0.0) | .06 | | |
|  | >0.6 to 4.3 |  | ˗0.1 (0.3) | ˗0.1 (˗0.7 – 0.4) | | .65 | |  | ˗0.3 (0.9) | | | ˗0.5 (˗1.4 – 0.5) | .30 | | |
|  | ≤0.6 (Ref ^d^) |  | 0 (0) | — | |  | |  | 0 (0.6) | | | — |  | | |
| **Within CAL^e^ limit** | |  |  | |  | | .21 |  | |  | |  | | .09 |  |
|  | >6.8 | 45 | ˗0.4 (0.4) | ˗0.1 (˗0.3 – 0.2) | | .55 | | 55 | ˗1.4 (1.5) | | | ˗0.6 (˗1.2 – ˗0.0) | **.04**^c^ | | |
|  | >6.1 to 6.8 |  | ˗0.2 (0.2) | 0.2 (˗0.1 – 0.4) | | .20 | |  | ˗1.0 (0.9) | | | ˗0.2 (˗0.7 – 0.4) | .59 | | |
|  | >4.1 to 6.1 |  | ˗0.3 (0.4) | 0.1 (˗0.1 – 0.3) | | .51 | |  | ˗0.7 (0.7) | | | 0.1 (˗0.5 – 0.7) | .71 | | |
|  | ≤4.1 (Ref^d^) |  | ˗0.2 (0.2) | — | |  | |  | ˗0.8 (1.5) | | | — |  | | |
| **Within CHO^f^ limit** | |  |  | |  | | .08 |  | |  | |  | | **.011**^c^ |  |
|  | >5.9 | 46 | ˗0.4 (0.3) | ˗0.1 (˗0.3 – 0.1) | | .30 | | 55 | ˗1.2 (1.5) | | | ˗1.0 (˗1.6 – ˗0.4) | **.001**^c^ | | |
|  | >4.3 to 5.9 |  | ˗0.1 (0.2) | 0.2 (0.0 – 0.4) | | .10 | |  | ˗1.4 (1.2) | | | ˗0.6 (˗1.2 – ˗0.0) | **.04**^c^ | | |
|  | >2.5 to 4.3 |  | ˗0.3 (0.4) | ˗0.1 (˗0.2 – 0.2) | | .62 | |  | ˗0.9 (1.1) | | | ˗0.7 (˗1.3 – ˗0.1) | **.03**^c^ | | |
|  | ≤2.5 (Ref ^d^) |  | ˗0.2 (0.2) | — | |  | |  | ˗0.2 (0.6) | | | — |  | | |
| **Choosing healthier food options** | | |  | |  | | .83 |  | |  | |  | | .21 |  |
|  | >4.3 | 46 | ˗0.2 (0.2) | ˗0.1 (˗0.3 – 0.2) | | .51 | | 54 | ˗1.2 (1.6) | | | ˗0.6 (˗1.2 – ˗0.0) | **.04**^c^ | | |
|  | >2.5 to 4.3 |  | ˗0.3 (0.4) | 0.0 (˗0.2 – 0.3) | | .79 | |  | ˗1.2 (1.1) | | | ˗0.3 (˗0.9 – 0.3) | .32 | | |
|  | >1.1 to 2.5 |  | ˗0.3 (0.2) | 0.0 (˗0.2 – 0.2) | | .91 | |  | ˗1.1 (1.0) | | | ˗0.2 (˗0.7 – 0.4) | .57 | | |
|  | ≤1.1 (Ref^d^) |  | ˗0.2 (0.4) | — | |  | |  | ˗0.5 (1.0) | | | — |  | | |
| **FBG^g^ measurement** | | |  | |  | | .87 |  | |  | |  | | **.002**^c^ |  |
|  | >1.3 | 67 | ˗0.2 (0.3) | ˗0.1 (˗0.3 – 0.1) | | .48 | | 93 | ˗1.3 (1.3) | | | ˗0.8 (˗1.3 – ˗0.2) | **.005**^c^ | | |
|  | >0.8 to 1.3 |  | ˗0.3 (0.3) | ˗0.1 (˗0.3 – 0.2) | | .59 | |  | ˗0.6 (1.1) | | | ˗0.4 (˗0.9 – 0.2) | .18 | | |
|  | >0.3 to 0.8 |  | ˗0.2 (0.3) | ˗0.1 (˗0.3 – 0.1) | | .54 | |  | ˗0.3 (0.9) | | | 0.1 (˗0.5 – 0.7) | .70 | | |
|  | ≤0.3 (Ref^d^) |  | ˗0.2 (0.3) | — | |  | |  | ˗0.4 (1.0) | | | — |  | | |
| **RBG^h^ measurement** | | |  | |  | | .24 |  | |  | |  | | **<.001**^c^ |  |
|  | >1.2 | 67 | ˗0.2 (0.2) | ˗0.1 (˗0.3 – 0.2) | | .57 | | 93 | ˗1.3 (1.4) | | | ˗1.2 (˗1.7 – ˗0.6) | **<.001**^c^ | | |
|  | >0.8 to 1.2 |  | ˗0.4 (0.4) | ˗0.2 (˗0.4 – ˗0.0) | | **.045**^c^ | |  | ˗0.6 (0.9) | | | ˗0.6 (˗1.2 – ˗0.1) | **.03**^c^ | | |
|  | >0.2 to 0.8 |  | ˗0.2 (0.2) | ˗0.0 (˗0.2 – 0.2) | | .70 | |  | ˗0.6 (0.9) | | | ˗0.6 (˗1.1 – 0.0) | .05 | | |
|  | ≤0.2 (Ref^d^) |  | ˗0.2 (0.3) | — | |  | |  | 0.1 (0.7) | | | — |  | | |
| **Weight charting** | | |  | |  | | **.04**^c^ |  | |  | |  | | **.002**^c^ |  |
|  | >3.8 | 67 | ˗0.3 (0.3) | ˗0.3 (˗0.5 – ˗0.1) | | **.009**^c^ | | 93 | ˗1.2 (1.3) | | | ˗1.0 (˗1.5 – ˗0.5) | **<.001**^c^ | | |
|  | >1.3 to 3.8 |  | ˗0.2 (0.2) | ˗0.2 (˗0.4 – 0.1) | | .12 | |  | ˗1.0 (1.0) | | | ˗0.7 (˗1.2 – ˗0.2) | **.005**^c^ | | |
|  | >0.8 to 1.3 |  | ˗0.1 (0.2) | ˗0.1 (˗0.3 – 0.1) | | .41 | |  | ˗0.7 (1.1) | | | ˗0.6 (˗1.0 – ˗0.1) | **.02**^c^ | | |
|  | ≤0.8 (Ref^d^) |  | ˗0.1 (0.3) | — | |  | |  | ˗0.1 (1.1) | | | — |  | | |
| **Achieving step count goal** | | |  | |  | | .27 |  | |  | |  | | **.010**^c^ |  |
|  | >3.2 | 67 | ˗0.2 (0.2) | ˗0.1 (˗0.3 – 0.1) | | .49 | | 93 | ˗1.3 (1.3) | | | ˗0.8 (˗1.3 – ˗0.3) | **.002**^c^ | | |
|  | >1.3 to 3.2 |  | ˗0.3 (0.4) | 0.0 (˗0.3 – 0.2) | | .67 | |  | ˗0.5 (0.9) | | | ˗0.3 (˗0.8 – 0.2) | .26 | | |
|  | >0.6 to 1.3 |  | ˗0.1 (0.3) | 0.1 (˗0.1 – 0.3) | | .30 | |  | ˗0.5 (1.2) | | | ˗0.1 (˗0.7 – 0.4) | .62 | | |
|  | ≤0.6 (Ref^d^) |  | ˗0.2 (0.3) | — | |  | |  | ˗0.5 (1.0) | | | — |  | | |
| **Communication with dietitian** | | | | |  | | .18 |  | |  | |  | | **<.001**^c^ |  |
|  | >4.4 | 67 | ˗0.3 (0.3) | ˗0.2 (˗0.4 – 0.0) | | .10 | | 93 | ˗1.1 (1.4) | | | ˗0.9 (˗1.4 – ˗0.4) | **<.001**^c^ | | |
|  | >3.0 to 4.4 |  | ˗0.3 (0.3) | ˗0.2 (˗0.4 – 0.0) | | .11 | |  | ˗1.2 (1.0) | | | ˗0.8 (˗1.3 – ˗0.4) | **.001**^c^ | | |
|  | >1.6 to 3.0 |  | ˗0.1 (0.3) | 0.0 (˗0.3 – 0.2) | | .72 | |  | ˗0.5 (0.7) | | | ˗0.4 (˗0.8 – 0.1) | **.10**^c^ | | |
|  | ≤1.6 (Ref^d^) |  | ˗0.1 (0.3) | — | |  | |  | ˗0.2 (1.1) | | | — |  | | |
| **Videos watched** | |  |  | |  | | .79 |  | |  | |  | | **<.001**^c^ |  |
|  | >14.1 | 67 | ˗0.2 (0.2) | ˗0.1 (˗0.3 – 0.1) | | .39 | | 93 | ˗1.0 (1.2) | | | ˗1.1 (˗1.6 – ˗0.6) | **<.001**^c^ | | |
|  | >7.0 to 14.1 |  | ˗0.3 (0.4) | ˗0.1 (˗0.3 – 0.1) | | .40 | |  | ˗0.9 (1.2) | | | ˗0.8 (˗1.2 – ˗0.3) | **.001**^c^ | | |
|  | >1.1 to 7.0 |  | ˗0.2 (0.3) | ˗0.1 (˗0.3 – 0.1) | | .44 | |  | ˗0.8 (1.3) | | | ˗0.6 (˗1.1 – ˗0.1) | **.01**^c^ | | |
|  | ≤1.1 (Ref^d^) |  | ˗0.2 (0.3) | — | |  | |  | ˗0.2 (0.8) | | | — |  | | |
| **Overall app utilization** | | |  | |  | | .67 |  | |  | |  | | **<.001**^c^ |  |
|  | >6.4 | 67 | ˗0.3 (0.4) | ˗0.1 (˗0.3 – 0.1) | | .42 | | 93 | ˗1.1 (1.3) | | | ˗0.9 (˗1.4 – ˗0.5) | **<.001**^c^ | | |
|  | >4.2 to 6.4 |  | ˗0.1 (0.2) | 0.0 (˗0.2 – 0.2) | | .88 | |  | ˗0.4 (0.8) | | | ˗0.4 (˗0.9 – 0.0) | .06 | | |
|  | ≤4.2 (Ref^d^) |  | ˗0.2 (0.2) | — | |  | |  | ˗0.3 (0.9) | | | — |  | | |
| **Number of app features with ≥75% uptake** | | |  | |  | | .30 |  | |  | |  | | .05 |  |
| ≥5  <5 | | 45 | ˗0.3 (0.3)  ˗0.2 (0.3) | | 0.1 (˗0.4 – 0.1)  — | | .30 | 54 | | ˗1.2 (1.5)  ˗1.0 (1.2) | | ˗0.6 ( ˗1.1 – 0.0)  — | | .05 |  |

^a^HbA_1c_: glycated hemoglobin.

^b^Adjusted for age, gender, and ethnicity and baseline HbA_1c_.

^c^Statistically significant *P* values when compared with reference quartiles.

^d^Ref: reference group.

^e^CAL: calorie.

^f^CHO: carbohydrate.

^g^FBG: fasting blood glucose; measured in the morning before food or water.

^h^RBG: random blood glucose; measured 2 hours following ingestion of breakfast, lunch, or dinner.
