# Supplementary material for: Association Between Mobile Health App Engagement and Weight Loss and Glycemic Control in Adults With Type 2 Diabetes and Prediabetes (D’LITE Study): Prospective Cohort Study
Source: JMIR Diabetes. 2022 Sep 30;7(3):e35039. doi: 10.2196/35039 (PMC9568822; doi:10.2196/35039)
Supplement: Multimedia Appendix 4 [file diabetes_v7i3e35039_app4.docx]

**Multimedia Appendix 4**

Associations between app engagement and HbA_1c_ change at 3 months for prediabetes and diabetes.

| App engagement  (number of days per week) | | **Participants with prediabetes** | | | |  | **Participants with diabetes** | | | |
| --- | --- | --- | --- | --- | --- | --- | --- | --- | --- | --- |
|  |  | Values, n | HbA_1c_ change from baseline (%),  mean (SD) | Mean difference  (95% CI) | *P* value^a^ | Values , n | HbA_1c_ change  from  baseline (%),  mean (SD) | | Mean difference  (95% CI) | *P* value^a^ |
| **Complete meal log** | |  |  |  | **.04^b^** |  |  |  | | **<.001^b^** |
|  | >5.9 | 68 | ˗0.3 (0.3) | ˗0.2 (˗0.5 – 0.0) | .11 | 95 | ˗1.0 (1.0) | ˗1.0 (˗1.4 – ˗0.5) | | **<.001^b^** |
|  | >4.1 to 5.9 |  | ˗0.2 (0.2) | 0.1 (˗0.1 – 0.3) | .59 |  | ˗1.1 (1.3) | ˗0.8 (˗1.2 – ˗0.3) | | **.001^b^** |
|  | >1.9 to 4.1 |  | 0 (0.2) | 0.2 (˗0.02 – 0.3) | .08 |  | ˗0.4 (0.7) | ˗0.2 (˗0.7 – 0.2) | | .30 |
|  | ≤ 1.9 (Ref^c^) |  | ˗0.2 (0.2) |  |  |  | ˗0.3 (0.7) |  | |  |
| **Any meal log** | |  |  |  | .15 |  |  |  | | **<.001^b^** |
|  | >6.9 | 68 | ˗0.2 (0.3) | ˗0.2 (˗0.4 – ˗0.0) | **.04^b^** | 95 | ˗1.2 (1.3) | ˗1.0 (˗1.4 – ˗0.6) | | **<.001^b^** |
|  | >6.3 to 6.9 |  | ˗0.1 (0.2) | ˗0.1 (˗0.3 – 0.1) | .19 |  | ˗1.1 (1.1) | ˗0.9 (˗1.3 – ˗0.5) | | **<.001^b^** |
|  | >3.8 to 6.3 |  | ˗0.2 (0.3) | ˗0.2 (˗0.4 – ˗0.0) | **.046^b^** |  | ˗0.7 (0.8) | | ˗0.5 (˗0.9 – ˗0.1) | **.008^b^** |
|  | ≤3.8 (Ref^c^) |  | 0 (0.3) |  |  |  | ˗0.1 (0.4) |  | |  |
| **Within CAL^d^ limit** | |  |  |  | .08 |  |  |  | | .11 |
| >6.7 | | 56 | ˗0.3 (0.3) | ˗0.1 (˗0.3 – 0.1) | .37 | 77 | ˗1.0 (1.1) | ˗0.6 (˗1.1 – ˗0.1) | | **.03^b^** |
| >6.0 to 6.7 | |  | ˗0.1 (0.2) | 0.1 (˗0.1 – 0.3) | .22 |  | ˗1.2 (1.1) | ˗0.3 (˗0.8 – 0.2) | | .26 |
| >4.0 to 6.0 | |  | ˗0.1 (0.2) | 0.1 (˗0.1 – 0.3) | .16 |  | ˗0.8 (1.1) | ˗0.1 (˗0.6 – 0.4) | | .77 |
| ≤4.0 (Ref^c^) | |  | ˗0.1 (0.2) |  |  |  | ˗0.5 (0.8) |  | |  |
| **Within CHO^e^ limit** | |  |  |  | .45 |  |  |  | | **<.001^b^** |
|  | >5.7 | 58 | ˗0.3 (0.3) | ˗0.1 (˗0.3 – 0.1) | .23 | 80 | ˗1.2 (1.2) | ˗1.0 (˗1.5 – ˗0.6) | | **<.001^b^** |
|  | >3.8 to 5.7 |  | ˗0.1 (0.2) | 0.0 (˗0.2 – 0.2) | .74 |  | ˗1.2 (1.3) | ˗0.8 (˗1.3 – ˗0.4) | | **.001^b^** |
|  | >2.2 to 3.8 |  | ˗0.2 (0.3) | ˗0.1 (˗0.3 – 0.1) | .24 |  | ˗0.7 (0.7) | ˗0.5 (˗1.0 – ˗0.1) | | **.02^b^** |
|  | ≤2.2 (Ref^c^) |  | ˗0.1 (0.3) |  |  |  | ˗0.3 (0.6) |  | |  |
| **Choosing healthier food options** | | |  |  | .98 |  |  |  | | **.007^b^** |
|  | >4.2 | 56 | ˗0.2 (0.3) | 0.0 (˗0.2 – 0.2) | .81 | 77 | ˗1.4 (1.4) | ˗0.8 (˗1.3 – ˗0.4) | | **.001^b^** |
|  | >2.3 to 4.2 |  | ˗0.2 (0.2) | 0.0 (˗0.2 – 0.1) | .88 |  | ˗1.0 (1.2) | ˗0.5 (˗0.9 – 0.0) | | .06 |
|  | >0.9 to 2.3 |  | ˗0.2 (0.3) | 0.0 (˗0.2 – 0.2) | .93 |  | ˗1.0 (0.8) | ˗0.5 (˗1.0 – ˗0.1) | | **.03^b^** |
|  | ≤0.9 (Ref^c^) |  | ˗0.1 (0.2) |  |  |  | ˗0.3 (0.6) |  | |  |
| **FBG^f^ measurement** | | |  |  | .08 |  |  |  | | **.007^b^** |
|  | >2.1 | 68 | ˗0.1 (0.1) | 0.0 (˗0.3 – 0.2) | .78 | 95 | ˗1.0 (1.1) | ˗0.6 (˗1.1 – ˗0.2) | | **.008^b^** |
|  | >1.3 to 2.1 |  | ˗0.3 (0.3) | ˗0.2 (˗0.4 – ˗0.1) | **.01^b^** |  | ˗0.8 (1.0) | ˗0.3 (˗0.8 – 0.2) | | .22 |
|  | >0.5 to 1.3 |  | ˗0.1 (0.2) | ˗0.1 (˗0.2 – 0.1) | .49 |  | ˗0.3 (0.9) | 0.0 (˗0.5 – 0.5) | | .90 |
|  | ≤0.5 (Ref^c^) |  | ˗0.1 (0.3) |  |  |  | ˗0.5 (0.7) |  | |  |
| **RBG^g^ measurement** | | |  |  | .06 |  |  |  | | **<.001^b^** |
|  | >2.1 | 68 | ˗0.2 (0.1) | ˗0.1 (˗0.3 – 0.1) | .34 | 95 | ˗1.2 (1.3) | ˗1.0 (˗1.5 – ˗0.5) | | **<.001^b^** |
|  | >1.3 to 2.1 |  | ˗0.3 (0.3) | ˗0.2 (˗0.4 – ˗0.1) | **.008^b^** |  | ˗0.7 (0.8) | ˗0.6 (˗1.2 – ˗0.1) | | **.01^b^** |
|  | >0.5 to 1.3 |  | ˗0.2 (0.2) | ˗0.1 (˗0.2 – 0.1) | .31 |  | ˗0.5 (0.8) | ˗0.4 (˗0.9 – 0.1) | | .11 |
|  | ≤0.5 (Ref^c^) |  | ˗0.1 (0.3) |  |  |  | ˗0.1 (0.5) |  | |  |
| **Weight charting** | |  |  |  | **.04^b^** |  |  |  | | **.03^b^** |
|  | >4.8 | 68 | ˗0.2 (0.3) | ˗0.1 (˗0.3 – 0.1) | .37 | 95 | ˗1.3 (1.2) | ˗0.7 (˗1.1 – ˗0.2) | | **.003^b^** |
|  | >1.8 to 4.8 |  | ˗0.2 (0.2) | 0.0 (˗0.2 – 0.2) | .77 |  | ˗0.9 (0.9) | ˗0.3 (˗0.7 – 0.1) | | .15 |
|  | >1.1 to 1.8 |  | 0 (0.3) | 0.1 (˗0.1 – 0.3) | .18 |  | ˗0.6 (0.7) | ˗0.2 (˗0.7 – 0.2) | | .32 |
|  | ≤1.1 (Ref^c^) |  | ˗0.2 (0.2) |  |  |  | ˗0.4 (1.0) |  | |  |
| **Achieving step count goal** | | |  |  | .19 |  |  |  | | **.001^b^** |
|  | >3.4 | 68 | ˗0.2 (0.2) | ˗0.2 (˗0.3 – 0.0) | .06 | 95 | ˗1.3 (1.3) | ˗0.6 (˗1.0– ˗0.2) | | **.005^b^** |
|  | >1.9 to 3.4 |  | ˗0.2 (0.2) | 0.0 (˗0.2 – 0.1) | .61 |  | ˗0.6 (0.7) | ˗0.2 (˗0.6 – 0.3) | | .41 |
|  | >0.9 to 1.9 |  | ˗0.1 (0.3) | 0.0 (˗0.2 – 0.2) | .85 |  | ˗0.3 (0.6) | 0.2 (˗0.2 – 0.7) | | .26 |
|  | ≤0.9 (Ref^c^) |  | ˗0.1 (0.2) |  |  |  | ˗0.7 (1.1) |  | |  |
| **Communication with dietitian** | | |  |  | .06 |  |  |  | | **.002^b^** |
|  | >4.7 | 68 | ˗0.2 (0.2) | ˗0.2 (˗0.4 – ˗0.06) | **.009^b^** | 95 | ˗0.9 (1.1) | ˗0.6 (˗1.0 – ˗0.2) | | **.008^b^** |
|  | >3.5 to 4.7 |  | ˗0.2 (0.2) | ˗0.2 (˗0.4 – ˗0.02) | **.03^b^** |  | ˗1.4 (1.2) | ˗0.8 (˗1.2 – ˗0.4) | | **<.001^b^** |
|  | >2.0 to 3.5 |  | ˗0.2 (0.3) | ˗0.2 (˗0.4 – 0.01) | .06 |  | ˗0.6 (0.9) | ˗0.3 (˗0.7 – 0.1) | | .17 |
|  | ≤2.0 (Ref^c^) |  | 0 (0.3) |  |  |  | ˗0.3 (0.6) |  | |  |
| **Overall app utilisation** | |  |  |  | .73 |  |  |  | | **<.001^b^** |
|  | >6.8 | 68 | ˗0.2 (0.3) | ˗0.1 (˗0.3 – 0.0) | .43 | 95 | ˗1.2 (1.3) | ˗1.0 (˗1.4 – ˗0.7) | | **<.001^b^** |
|  | >5.5 to 6.8 |  | ˗0.1 (0.2) | 0.0 (˗0.2 – 0.1) | .64 |  | ˗0.4 (0.6) | ˗0.4 (˗0.8 – ˗0.0) | | **.03^b^** |
|  | ≤5.5 (Ref^c^) |  | ˗0.1 (0.2) |  |  |  | ˗0.3 (0.6) |  | |  |
| **Number of app features with ≥75% uptake**  ≥5 | | 56 | ˗0.3 (0.3) | ˗0.1 (˗0.3 – 0.1) | .16  .16 | 77 | ˗1.5 (1.5) | ˗0.8 (˗1.4 – 0.1) | | **.02^b^**  **.02^b^** |
| <5 | |  | ˗0.1 (0.2) |  | |  | ˗0.8 (1.0) |  | |  |

^a^Adjusted for age, gender, ethnicity, and baseline HbA_1c_.

^b^Statistically significant *P* values when compared to reference quartiles.

^c^Ref: Reference group.

^d^CAL: Calorie.

^e^CHO: Carbohydrate.

^f^FBG: Fasting blood glucose measured in the morning before food or water.

^g^RBG: Random blood glucose measured two hours following ingestion of breakfast, lunch or dinner.
